# Kazin’s Trilling: A Cold War Portrait

**DOI:** 10.1007/s12115-018-0301-7

**Published:** 2018-11-05

**Authors:** Richard M. Cook

**Affiliations:** College of Arts and Sciences, Department of English, University of Missouri-St. Louis, 461 Lucas Hall, 1 University Blvd, St. Louis, MO 63121-4400 USA

**Keywords:** Alfred Kazin, Lionel Trilling, Diana Trilling, Walt Whitman, Matthew Arnold, Cold War, Autobiography, *New York Jew*, Class and culture

## Abstract

This essay describes the eminent Americanist, critic, and New York intellectual, Alfred Kazin’s creation of a Lionel Trilling “character” in his 1978 autobiography, *New York Jew*, and his use of that character to critique significant features of the country’s Cold War literary culture. Among these are: the narrowing and hardening of intellectual discourse in a cultural-political climate dominated by the “liberal consensus,” the discrediting of the progressive impulse in American writing, the subordination of “class” to “culture” in evaluations of American writers, and the changing status of Jews and Jewish writers in post-war America. Tapping into strong personal feelings, Kazin creates in Trilling a harsh, thoughtful and compelling portrait of an era.

'I feel I do not belong to any of it." Alfred Kazin, journal entry (6/27/55)

In May of 1978 Alfred A. Knopf published *New York Jew,* the third autobiographical work by the eminent literary critic and Americanist, Alfred Kazin. Kazin’s previous autobiographies (or memoirs or “personal histories” as he called them), *A Walker in the City* (1951) and *Starting Out in the Thirties* (1965) describing his Brooklyn childhood and his early successful efforts to become a writer, had received very strong reviews, many of which took note of the author’s gift for vivid, shrewd literary portraiture. Thus expectations were high for his third memoir billed as an insider’s account of the post-war literary-intellectual world in which Kazin was still a prominent figure. It did not disappoint. *New York Jew* should “give pleasure to anybody interested in the literary history of our times,” wrote Mordechai Richler on the front page the *New York Times Book Review*. “But it should also make the viewer wonder at what self-serving rascals most of our literary heroes are.” Richler was exaggerating. There are admiring, affectionate portraits of Edmund Wilson, Hannah Arendt, and Saul Bellow. But it was the negative ones, particularly the surprisingly harsh portrait of the recently deceased (1975) Lionel Trilling that received the most attention, raised the most questions, and provoked the most consternation. Why would Alfred Kazin, one of the country’s best known and respected public intellectuals feel the need to create such a scathing portrait of a revered cultural figure with whom he apparently had no on-going public quarrel?

In fact, there had been serious differences and difficulties between them as far back as the early forties, differences that did not produce a sustained public debate but that had long troubled Kazin and that extended into the culture at large. A look back at the portrait and some relevant entries from both writers’ diaries together with a brief review and discussion of their relationship (or non-relationship) will indicate some of the personal and political sources of Kazin’s difficulty with Trilling and why he chose to make him a pivotal figure in his chronicle of the post-war years. They also shed new light on some of the personal/ideological tensions and disagreements at work in the forties and early fifties that rarely broke the surface of the so-called Cold-War “liberal consensus.” Staunchly anticommunist and centrist in its politics, that consensus, perhaps more accurately labeled “liberal conservatism,” discouraged the kind of intellectual dissent (including serious discussions of socialism) that had characterized the pre-war and progressive years (Hodgson 1976, 72). Public dissenters and would-be dissenters like Kazin risked being seen as irresponsible or ignorant--if they were heard at all. Writing *New York Jew* was Kazin’s latter-day effort to be heard, to place on the record his thoughts about the Cold War years and, more specifically and personally, his feelings about a writer whom he admired, distrusted, and resented and who more than any other single figure shaped the literary culture of an era he (Kazin) had found constricting, frustrating, and alien -- “I feel I do not belong to any of it” (*Journals*, 6/27/55).

The portrait comes in two installments. The first describing Kazin’s initial impressions of Trilling begins positively. He recalls Trilling’s “warm” review of his literary history, *On Native Ground*s (1942), and adds that “Trilling was an intense intellectual admiration of mine.” Anticipating later difficulties, he follows up with the observation that Trilling, who was ten years his senior, “had absorbed the more gentlemanly style of the twenties much as [Saul] Bellow and I had absorbed the social angers of the abrasive lower-class thirties” (43). He notes Trilling’s good looks and graceful public manner, but he also sees them as a form of disguise—“white hair over a handsome face that seemed to be furrowed, hooded, closed up in constant thought. The life was all within, despite his debonair practiced easiness of manner” (42). Kazin found the discrepancy fascinating and disconcerting: “There was an immense and even cavernous subtlety to the man, along with much timidity, a self-protectiveness as elegant as a fencer’s.” He concludes his early impressions with a mocking characterization of their first meeting in 1942 in the offices of the *New Republic*, which had recently hired Kazin as literary editor. After a brief conversation about possible books to review, “Trilling astonished me by saying, very firmly, that he would not write anything that did not ‘promote my reputation.’” Such “solemnity” about his reputation struck Kazin as “hilarious.” “It seemed to resemble an expensive picture on view. ‘My reputation’ was to be nursed along like money in the bank. It was capital. I had never encountered a Jewish intellectual so conscious of social position, so full of adoptive finery in his conversation” (43). Kazin’s Trilling may be highly intelligent, subtle and handsome, but he is also defensive, ambitious, vain, and affected—a cultural snob. Moreover, he is a reluctant, invisible Jew, “this extraordinarily accomplished son of an immigrant [Jewish]tailor” who “quietly defended himself from the many things he had left behind” (43).

In a later chapter, “The Times Being What They Are,” describing life during the McCarthy years, the portrait shifts to Trilling’s role in the Cold War—“Trilling’s high moment” (191). A one-time radical, who in the 1930s had been fiercely critical of America, Trilling became “the most successful leader of deradicalization” in the post war years” and an enthusiastic “convert [to]America as an ideology” versus the Soviet Union (190–1). Yet, he and those influenced by him were largely uninterested in what was happening at home-- on the shop floor, in the board rooms, in the segregated South, and especially on the streets of New York. Like his mentor, Matthew Arnold, Trilling wrote “as if the only problem of society was the thinking of the ‘advanced intellectuals’” (192). In Trilling’s America “there were no workers, nobody suffering from a lack of cash; no capitalists, no corporations, no Indians, no blacks. And on the whole neither were there any Jews” (192). Instead there were “cultured minds, “intellectual contentment,” and “middle-class claustrophobia,” as well as a notable reluctance to espouse or defend views that could be interpreted as a challenge to the “New Liberal” consensus that anti-Stalinism must constitute the bed-rock of all serious intellectual discourse. It was a time when “it seemed impossible in public to admit doubts, divisions, nuances, contradictions, hesitations, lost illusions. . . . ‘Radical’ was not mentioned. ‘Communist’ could be applied only to those who could not confess their past” (190–2).

Not surprisingly, the portrait provoked a hostile response from Trilling’s friends and colleagues who in letters to the *New York Times* called it a grotesque misrepresentation of the truth and from reviewers who accused Kazin of settling old scores. Kazin was unrepentant. “There is not a single line about Trilling I would take out of my book or apologize for,” he wrote in a journal entry (*Journals*, 6/26/78). He had had his say on a subject that had been troubling him for years—not through debate but by creating a Trilling “character.” “Trilling, the pompously respectable professor, is a character in *my* imagination of society, not a person to argue with. . . . I must *use* my inner resentments as possibilities for literary use” (*Journals,* 3/5/66). Which is what he did—and more. The Trilling “character” was both an expression of personal grievance and an opportunity to focus attention on long-standing matters of contention in Trilling’s Cold War America, among them: the narrowing and hardening of intellectual-political discourse, the discrediting of the progressive impulse in American writing that Kazin had celebrated in *On Native Grounds*, the subordination of “class” to “culture” in discussions and evaluations of American writers, and the changing status of American Jews in the post-war years. These were not the only factors shaping relations with Trilling: incompatible temperaments, evident in Trilling’s personal diary discussed below, were perhaps even more determinative. But they mark significant differences that extend beyond personal disputes into the post -war culture. Kazin may have opted for “character” over argument, but character can serve the purposes of argument. Kazin was not just talking back to Trilling in *New York Jew*; he was talking back to post-war America.

Kazin’s relationship with Trilling began affably enough with a favorable review of *On Native Grounds* —though events leading up to the review suggest difficulties to come. Kazin had begun his history in 1938 following a suggestion from the self-described “progressive” Americanist Carl Van Doren, who had been reading his reviews in the New York papers (Van Doren 1936, 9). In a 1939 (successful) application for a Guggenheim grant, Kazin, who shared Van Doren’s progressive views made clear his intentions for the book, originally titled, *The Years of Promise: Prose in America Since 1900*. He would demonstrate that recent American writers, following the 1907–1914 progressives, realists, and naturalists, were advancing a national literature that “has always been a social and political literature.” Acknowledging that writers in the twenties had turned from reform to “disillusionment,” he would show that “the 1930s marked a return to the sense of responsibility.” He concludes the application with a tribute to Vernon Parrington, whose progressive history, *Main Currents in American Literature* (1927), he claims was “the inspiration” for his own history. “It was Parrington who first taught me how moving the homely, passionate idealism of American literature can be. . . . It was Parrington who taught me to regard American literature as a democratic epic whose Homers have been economists as often as they have been poets, philosophers talking the language of the good society, historians with the sensibility of imaginative artists.”

Four months after he submitted his application *Partisan Review* published a fierce attack on Parrington by Trilling, who argued that his simplistic view of reality devalued the achievement of the country’s best writers and demonstrated a fundamental failure of mind. Kazin took notice. The Parrington who appears in *On Native Grounds* in 1942 is a much diminished figure while Trilling is praised for setting the record straight. “One cannot help wondering, as Lionel Trilling has said so well, ‘if the literary historian who does not have the perception to understand our best artists understands more than our simplest problems’” (*Grounds,*163). On its publication Kazin’s history was widely and favorably reviewed--by Trilling among others: “quite the best and most complete treatment we have of an arduous and difficult subject” (“Four,” 483). Trilling makes no mention of Parrington or of his 1940s attack, but he could only have been gratified by Kazin’s second thoughts about *Main Currents*.

Kazin’s next critical encounter with Trilling came more than a decade later in a 1954 essay introducing a collection of essays on Theodore Dreiser. This time there were no second thoughts. Dreiser had long been a Kazin favorite. His discussion of the novelist in *On Native Grounds* stands out even today for the depth its critical sympathy. Thus his understandable dismay when he read Trilling’s attack on Dreiser in the April 1946 issue of *Partisan Review*. While Trilling’s real target seems to have been the liberals and progressives who he felt overlooked Dreiser’s faults because they approved of his politics, he wanted it understood that they were indulging a writer notable *for* his faults:—his “dullness,” “stupidity,” “foolishness,” anti-Semitism,” and, of course, his “vulgarity” (*Liberal*, 10–21). Kazin was angry and disgusted. “I hated what Trilling wrote about Dreiser,” he write in a letter to me. “It was so snobbish and so overtly political.” He was only slightly more measured in his 1954 Dreiser defense. Instead of engaging with Dreiser’s novels, Trilling, according to Kazin, has turned him into a symbol of Stalinoid liberalism. “What happens whenever we convert a writer into a symbol is that we lose the writer himself in all his indefeasible singularity” (*Dreiser,* 10). “Literary people as a class can get so far away from the experience of other classes that they tend to see them only symbolically. Cultivating “a certain genteel uninvolvement,“ they encourage us to forget “Dreiser’s breadlines and street-car strikes. . . . So, now we are ashamed of him because he brings up everything we should like to leave behind us” (10).

By the time of Kazin’s response to the attack on Dreiser Trilling’s political/cultural intentions had been long been evident. His 1940 piece on Parrington was but the first foray in a successful counter-progressive campaign against the country’s realist-naturalist, socially oriented literary tradition to which Kazin had devoted his literary history and which still held his sympathy. Also clear, following the 1951 publication of *The Liberal Imagination*, was Trilling’s disdainful attitude toward much, not all, of American fiction. This collection of previously published pieces included charges against Parrington, Dreiser, Dos Passos, Thomas Wolfe, Sherwood Anderson (another Kazin favorite), and Trilling’s celebrated 1948 essay, “Manners, Morals and the Novel,” in which he slights Faulkner for his provincialism, quotes approvingly Henry James’s “cogent” remarks on the items necessary for the novel missing from the American landscape, observes that the American’s resistance to considerations of “class” and “manners” leaves their novelists without the material on which the “classic” novel depends, and argues that if the democratic impulse has broadened our “social sympathies,” it has done so at the expense of “our power to love, for our novels can never create characters who truly exist” (217). Trilling’s counter-progressive arguments and his critique of the American novel would prove extraordinarily influential leading to what has been called a “paradigm revolution” in the critical-scholarly assessment of American literature (Wise 1973, 223–296). For Kazin, struggling to write a new “Preface” for *On Native Grounds* that he feared would make him appear “ridiculous,” they confirmed his sense of being stranded in an alien zeitgeist, and his growing conviction that there was an “irremediable opposition” between himself and Trilling and that “nothing whatever can be done about this” (*Journals*, 11/8/51).

That opposition, or Kazin’s feelings of opposition, derive in no small part from his disdain for the cultural role Trilling had carved out for himself as the mentor to what he (Trilling) repeatedly referred to as “the educated class” or “the reading class.” In his Dreiser piece, Kazin laments the separation dividing “literary people” from the “other classes.” Whether or not Kazin’s “literary people” qualify as a “class,” they constitute a group whose interests are not those of the other classes, including presumably those of the working class. It is this group of “literary people” and their “genteel uninvolvement” that Kazin identifies with Trilling-- Trilling, who championed Henry James over Dreiser at the “dark and bloody crossroads where literature and politics meet” (*Liberal*, 11); who turned “the great world of the English nineteenth century novel. . . into a personal dream” (*Jew*, 192); and who convinced or deluded himself, as Kazin would have it, into believing that the second coming of Matthew Arnold as Lionel Trilling would somehow make the difference between culture and anarchy in twentieth century America. “The cultural lag here is stupefying,” Kazin wrote in his journal after reading Trilling’s *New York Times* obituary: “The Jew totally mesmerized by a culture that no longer exists” (*Journals,*11/7/75). He had said much the same nine years earlier: “No one was ever so much the prisoner of culture as Trilling. No one was ever so much the victim of the genteel fantasy” (*Journals*, 3/5/66).

Heated discussions of class, culture, and gentility go back in American literary history at least as far as Whitman. “The word of the modern,” Whitman wrote in *Democratic Vistas* is: “Culture.” “Culture, we find ourselves abruptly in close quarters with the enemy. . . . As now taught, accepted and carried out, are not the processes of culture rapidly creating a class of supercilious infidels” (Whitman 1959, 479)? The effect on American society, Whitman argued, was to create and deepen class divisions. The effect on the “cultured” individual was to turn him into a “thoroughly upholstered exterior appearance and show, mental and otherwise, built entirely on the idea of caste” (478–9).

What would Whitman have thought of Lionel Trilling? One cannot be sure, but he might well have regarded him in terms similar to those directed at Trilling’s hero, Matthew Arnold. Arnold, Whitman complained, “brings to the world what the world has a surfeit of.. . delicacy, refinement, elegance, prettiness, propriety, criticism, analysis: all of them things which threaten to overwhelm us” (quoted in Raleigh, 60). Arnold “is one of the dudes of literature” (61). Kazin had a much higher opinion of Arnold, who, he once claimed, was responsible “for some of the greatest examples of [passionate] criticism in modern times” (*Contemporaries*, 496). And he had a high opinion of Trilling’s book on Arnold. But like Whitman he worried about the influence of British “snottiness” on American literary opinion (*Journals,* 3/24/66). And like Whitman, he was impatient and at times deeply suspicious of all the talk about this modern word “culture.” “Barzun, Trilling,” he wrote in a 1974 entry, “the endless prating of ‘culture.’ They mean not works of art but a vaguely synoptic tradition in space and time—the cultural continuum of the upper classes (the non-desperate classes)” (*Journals,* 12/5/74).

The fact that Trilling was a Jew only heightened Kazin’s scorn for his self-appointed role as cultural mentor for the “educated class.” Kazin was pleased to credit the many “brilliant Jews” including Trilling who had distinguished themselves as “immensely subtle and learned authorities” on Matthew Arnold, Henry James, James Joyce, Paul Cezanne (44). But he saw something phony and snobbish in Trilling’s Anglophilia and his effort to turn himself into an American Matthew Arnold – “the Jew’s dream of literary England, of surpassing his servile state by culture” (*Journals* 3/5/66). Trilling’s England was not only a dream; it was also, in Kazin’s view, part of a pattern of denial, even of repudiation—of working class America; of America itself; of New York City, which he merely “tolerated”; of “Jewishness,” --“to be a Jew and not be Jewish”-- and, more personally, of Kazin himself. (*Jew*, 191; 44). “For Trilling I would always be ‘too Jewish,’ too full of my lower-class experience. . . . . This would go on for thirty years, it *was* the barrier, like his fondness for the words ‘scarcely,’ ‘modulation,’ ‘our educated classes.’ I had scarcely enough modulation” (46–47).

Reviewers correctly criticized Kazin for calling Trilling an inauthentic Jew and for presenting himself as the genuine article spurned for being “too Jewish.” It was presumptuous to claim oneself as the measure of true Jewishness, a standard that Trilling had apparently failed to meet. He was also wrong to imply, as he does, that Trilling barred him from the Columbia English Department because he was a Jew. Over the years, Trilling supported the Department’s hiring a number of Jews. But Kazin was not wholly wrong about Trilling’s view of Jews who seemed to him too Jewish, or at least too demonstrably Jewish—“the peculiarly Jewish demonstrativeness, the showing of that which is within, the ghastly over-personalization of life” -- Trilling wrote in an October 10, 1952 journal entry reflecting on his distaste for Jews who appeared to flaunt their Jewishness in a play for unwelcome intimacy.^1^ In another entry, after noting that one thing that Kazin and Bellow have in common is their “antagonism to me,” he found it predictable that they sympathized with the “sappy-pretentious quality of [Isaac Rosenfeld’s] work. . . the self-pity, the conscious ‘Jewishness,’ . . . the exhibitionism” (4/20/67). One can readily imagine what Trilling would have thought of the demonstrative title of Kazin’s last memoir.

All three of Kazin’s memoirs are consciously and self-consciously Jewish—“my autobiography will always be most deeply the autobiography of a Jew,” he wrote in a 1960 entry (*Journals*, 1/4/60). However, unlike the earlier works that tell of Kazin’s youthful aspirations and efforts to break into the New York publishing world, *New York Jew* begins with success, with the enthusiastic reception of *On Native* Grounds, before proceeding to chronicle further personal successes—even as it challenges what such success means. For Kazin looking back, success had a serious downside: no more youthful yearning for the big break, the big book that would change everything: “I’ve had such as sense of looking forward to nothing, of having made it,” he wrote in a 1960 entry. “I want in my heart, obviously, to be an initiate again, to write the story of youth, to look forward to everything “(*Journal,* 5/13/60). For Jews generally, their spectacular post-war success in rising into the middle-class and joining the American mainstream meant it would be more difficult to claim their traditional status as America’s “archetypal minority” (Biale 1998). They were now part of the “white” majority, while other groups--Blacks, Latinos, Native Americans — were asserting their special difference—“the most marvelous cacophony,” Kazin wrote in a 1970 entry, “singing away in the cultural pond.” One intent of *New York Jew* in a period of growing multiculturalism was to remind Jews of their historical difference—the Holocaust is cited repeatedly—and to insist that they take a resolute, even a “demonstrative” stand, on their Jewishness. Singling out and characterizing Trilling as a non-Jewish Jew—“to be a Jew and yet not be Jewish” -- who objected to Kazin’s being “too Jewish” nicely suited the purpose (44).

Differing literary preferences, counter-progressive thinking, conflicting class loyalties, and sharply opposing attitudes about Jewishness are all at work in the Trilling portrait; but its sustained bitter tone suggests a source of anger and personal hurt more intractable than arguable differences. Kazin was painfully sensitive to slights and snubs, real and imagined. “No doubt there is still a lot of self-conscious, proletarian youth in me,” he wrote in a 1983 entry wondering if there was a party somewhere in progress to which he had not been invited. There may well have been. Though he could be charming, witty, and sympathetic, depending on his mood, Kazin was known to be a difficult person. Some of the snubs were likely real.

Early in *New York Jew* he describes a strained evening at the Trillings after which he was never invited back. He blamed Diana Trilling for poisoning the evening and later “for the public and humiliating exclusion of me from literary gatherings over which she presided” (April 16, 1997 letter to me). But Lionel also found Kazin a disquieting presence as his remarks on his “demonstrative” Jewishness indicate. And there may have been other reasons. “Lunch with Alfred Kazin,” he wrote in a 1947 diary entry. “Recollection of the ambivalence he generates proved perfect—the same flirtatiousness, the same real interest in one, the daring movement toward intimacy—you consent, are involved, then draw back repelled.” Trilling clearly found conversation with Kazin disturbing—and for a seemingly sexualized reason: he sensed that he was being seduced and felt himself yielding before drawing back repelled. Despite the language of flirtation and intimacy, it is not clear that Trilling saw the incident as a homosexual advance. His wife, however, had little doubt. Kazin’s “book will be history,” she complained to a friend about *New York Jew* shortly after its publication. It “will become the truth.. .. Nobody will know.. . the real truth.. . that Alfred had deep homosexual feelings for Lionel. Anybody who reads the book should be able to feel that, but who will?” (*Lerman*, 437).

I find no evidence in *New York Jew* or his journals that Kazin had homosexual feelings for Trilling—though, like a number of Trilling’s students and younger writers, he may have looked to Trilling for intellectual and emotional support. There is, however, abundant evidence that Kazin was attracted to writers with an intensity some may have found unnerving: “Strange to lie in bed this morning, meditating on a discussion I had had in a dream with Trilling and to realize how real is the relationship I have with writers I have rarely or never met,” he wrote in a 1946 entry. “I have intricate entanglements with writers whom it always shocks me to meet—so real have their thoughts been to me, and so pressing the amount of agreement, instruction and divergence” (*Journals,* 10/3/46). Kazin liked writers, liked reading them, meeting them and assessing them. He prided himself, with some justification, on his ability to “read the mind behind each book,” to identify with the writer while maintaining critical distance (*Jew,* 8). Does this suggest a homoerotic element in Kazin’s personal encounter with Trilling? It seems more likely that he was looking for “agreement, instruction and divergence” and perhaps even a shot at a position at Columbia.

In a January 1974 letter to his editor at Little, Brown, Kazin wrote that *New York Jew* like his other memoirs should be read as a “personal history” –“the historic in the personal and the personal in the historic.” In other words, it was *his* history, and he made no claims that it was objective, fair, and balanced—though he clearly felt that a personal account of “a life lived among brilliant intellectuals” would be of interest and would make its own contribution to history (*Jew,* 42). The Trilling portrait is an essential part of this contribution, a minority report, perhaps, admittedly driven by resentments put to “literary use.” It is also a bitter reflection on Cold-War culture, personified in the Trilling “character,” who Kazin saw as an agent, beneficiary, and symbol of the “New Liberal” orthodoxy. Kazin had spoken out from time to time in the late 40`s and 50`s about the critical distortions attributable to this orthodoxy, including a devastating critique of (Trilling protégé) Richard Chase’s *Herman Melville: A Critical Study* (1949) for attempting to locate Melville in the “new liberal,” anti-Stalinist camp. (“Scripture,” 197). But, being an uneasy and reluctant polemicist, he did not launch the kind of frontal assault on conservative trends that marked Irving Howe’s 1954 attack on “This Age of Conformity”; instead, he began work (in 1955) on a new autobiographical effort that would trace the rise and transformation of “the beggarly Jewish radicals of the 1930s [into] the ruling cultural pundits of American society” (*Journals,* 7/25/1963). It would be his own story; but in the telling he could write about “the ex-left, about [Leslie] Fiedlerism, and [Sidney] Hookism, and Trillingism,” about the retreat from radical and progressive idealism; and he would do so on his own terms, in his own “brash” language.^2^ *New York Jew* is a consciously brash, impolite book. There is little of the “modulation” – “the tremulous carefulness and deliberation” that Kazin found in Trilling’s prose (193).^3^ Like the book itself and its title the Trilling portrait is defiant and irreverent. It may even have persuaded some readers, particularly those who like Kazin who had found Cold War culture intellectually narrow and stultifying. The portrait did not, to be sure, become “the truth” as Diana Trilling feared. It did, however, provide an arresting and iconoclastic view of a lionized figure –a Trilling “character” enabling Kazin to come to terms with his long anguished feelings towards a writer he admired and resented while reviewing and assessing his important role in the country’s Cold War literary culture.

## Footnotes

1 Trilling’s first published story in the *Menorah Journal* describes the narrator’s defensive response to a “scrubby little Jew.. . who is too much of my own race” and who “wants to tell [me] things about his soul.” “I do not want to know about people’s souls.. . I was particularly reluctant to see this person’s insides; they would be, probably, too much like mine.” “Impediments.” *Menorah Journal* 11 (June 1925): 286–299.

2 The proposed book would turn into two books: *Starting Out in the Thirties* (1965), and *New York Jew* (1978).

3 In an August 18, 1955 journal entry, Kazin comments on what he sees as the inauthentic or “specious” quality of Trilling’s prose. “I suppose that what it is I dislike most in Trilling’s writing is this specious air of reasonableness which he gives to his fears and prejudices; this air of connecting logic which makes so many of his points seem external. Compare it with the way Wilson or Malraux writes and you see the difference between someone (the latter), who writes straight from within, who is feeling his thoughts, rather than someone who is trying to think out what he is supposed to feel, or would like to show that he feels, rather than what he actually does. Trilling’s writing is so completely audience-minded, in this sense; it is the style of a lecturer, whose very turns and pauses and connecting phrases show that he is thinking of the audience.
